# Pim-2 Kinase Regulates Energy Metabolism in Multiple Myeloma

**DOI:** 10.3390/cancers15010067

**Published:** 2022-12-22

**Authors:** Zhaoyun Liu, Yixuan Guo, Xiaohan Liu, Panpan Cao, Hui Liu, Xifeng Dong, Kai Ding, Rong Fu

**Affiliations:** Department of Hematology, Tianjin Medical University General Hospital, Tianjin 300052, China

**Keywords:** Pim-2, multiple myeloma, metabolic flux, oxidative phosphorylation, cancer marker

## Abstract

**Simple Summary:**

This study reveals the role of Pim-2 kinase in regulating the metabolism and proliferation of multiple myeloma (MM) cells. We believe that our study makes a significant contribution to the literature because, using patient data as well as cell line-based metabolomic studies, we establish that Pim-2 kinase directly regulates glycolysis, energy production, and survival and that Pim-2 is a potential target for MM treatment.

**Abstract:**

Pim-2 kinase is overexpressed in multiple myeloma (MM) and is associated with poor prognosis in patients with MM. Changes in quantitative metabolism, glycolysis, and oxidative phosphorylation pathways are reportedly markers of all tumor cells. However, the relationship between Pim-2 and glycolysis in MM cells remains unclear. In the present study, we explored the relationship between Pim-2 and glycolysis. We found that Pim-2 inhibitors inhibited glycolysis and energy production in MM cells. Inhibition of Pim-2 decreased the proliferation of MM tumor cells and increased their susceptibility to apoptosis. Our data suggest that reduced Pim-2 expression inhibits the energy metabolism process in MM, thereby inhibiting tumor progression. Hence, Pim-2 is a potential metabolic target for MM treatment.

## 1. Introduction

Pim-2 kinase, a member of the Pim family constituting a class of serine/threonine kinases [1], is encoded by the oncogene Pim-2, located on the X chromosome [2], and is highly expressed in multiple cancers, including most hematological malignancies (leukemia, myeloma, and lymphoma) and some solid tumors (liver, lung, prostate, and breast cancers) [3,4,5]. Increased PIM kinase expression is related to the progression and prognosis of diseases, making it a potential biomarker candidate and therapeutic target. 

Multiple myeloma (MM), a cancer of plasma cells, occurs mainly in the bone marrow. Despite several new treatment options, it is still considered incurable. Malignant proliferation of monoclonal plasma cells causes anemia. MM cells not only provide a cellular microenvironment to protect themselves from the immune system [6] but also produce a variety of cytokines to stimulate osteoclastogenesis and suppress osteoblastic (OB) differentiation from bone marrow stromal cells, leading to extensive bone destruction [7].

Pim-2 kinase levels were significantly higher than those of other Pim family members in CD138^+^ myeloma cells in a series of 41 patients with newly diagnosed MM, suggesting that Pim-2 is relevant to MM cell proliferation [8]. Several studies have identified that Pim-2 promotes MM cell proliferation and inhibits apoptosis via the IL-6/STAT3 and NF-κB pathways, respectively [6,9,10,11]. Abnormal cell cycle in MM can be regulated by Pim-2 kinase by maintaining the phosphorylation of critical mediators, including p21Cip1/Waf1 (CDKN1A) [12] and p27KIP1 (CDKN1B) [13,14]. Pim-2 kinase is also a common downstream mediator for the suppression of osteoblastogenesis in myeloma [15], suggesting that it plays a role in bone metabolism [16]. However, few experimental studies have addressed the metabolic profile of myeloma cells, and whether Pim-2 kinase plays a role in the metabolic processes of myeloma cells remains unknown. 

Our experiments revealed that the level of Pim-2 kinase increased in MM patients and was related to energy metabolism. Moreover, mass spectrometry-based untargeted metabolomics indicated that energy metabolism participates in the progression of myeloma. We also report that inhibitors of Pim-2 kinase prevented cell growth and ATP production by disrupting glycolytic flux and oxidative phosphorylation. Further, inhibitors of Pim-2 kinase could also regulate glycolytic enzymes and the adenosine monophosphate-activated protein kinase (AMPK) pathway involved in energy metabolism.

## 2. Materials and Methods

### 2.1. Patient Information

All patients were treated at the Department of Hematology, Tianjin Medical University General Hospital, from September 2020 to September 2021. The patient group consisted of 23 newly diagnosed MM (NDMM) patients and 30 healthy controls (Table 1). The Human Ethics Committee of the Tianjin Medical University General Hospital approved the research protocol. Various characteristics of the patients are listed in Table 1.

Bone marrow samples were collected from patients as described previously. Patient bone marrow samples were centrifuged to obtain the supernatant, and the samples were stored at −80 °C for subsequent LC-MS/MS analysis. To keep the concentration of metabolites consistent in each group, we took 200 μL of supernatant from each group for the assay.

### 2.2. Cell Culture

Human myeloma cells (MM1. S, U266, and OPM2) were purchased from the Tumor Cell Bank of the Chinese Academy of Medical Sciences (Beijing, China). The cell lines were cultured in RPMI 1640 (Gibco, Life Technologies, Carlsbad, CA, USA) containing 10% fetal bovine serum (FBS) (Gibco, Life Technologies, Carlsbad, CA, USA) and 1% penicillin/streptomycin (Solarbio, Beijing, China) in an incubator at 37 °C in a humidified atmosphere consisting of 95% air and 5% CO_2_. The Pim kinase inhibitor SMI-16a was provided by MedChemExpress (Monmouth Junction, NJ, USA) and was frozen as a stock solution at −80 °C.

### 2.3. Metabolomic Analysis

We selected MM1.S, U266, and OPM2 cells as MM cell lines and divided them into control groups as well as a Pim-2 inhibitor-treated group. We collected 1 × 10^7^ cells per group to ensure homogenization. After collecting the suspended cells, they were washed with ice-cold PBS and centrifuged at 900 rpm in triplicates. The cells were quickly placed in liquid nitrogen for 30 s and stored at −80 °C for LC-MS/MS analysis. LC-MS/MS analysis was performed using an ultra-performance liquid chromatography (UHPLC) system (Thermo Fisher Scientific, Waltham, MA, USA) with a UPLC BEH amide column coupled to a Q-exactive HFX mass spectrometer. Electrospray ionization was used to test for both positive and negative ions. The qualitative and quantitative results of the metabolome were subjected to univariate and multivariate analyses (MVA) to screen for metabolites that appeared significantly different.

### 2.4. Cell Viability Assay

The MM cells were harvested and seeded in 6 well plates. Next, 10 μL of CCK-8 reagent (CCK-8; Bimake, Houston, TX, USA) was added to the wells, which were placed in an incubator at 37 °C in a 5% CO_2_ atmosphere for 3 h, and then the absorbance was read at 450 nm using a microplate reader. The cell viability percentage was calculated as follows:

Cell viability (%) = [a (trial group) − A (blank group)]/[a (control group) − A (blank group)] × 100. A (blank group): absorbance of wells with medium and CCK-8 solution without cells; a (trial group): absorbance of wells with cell, CCK8 solution, and drug solution; and a (control group): absorbance of wells with cells and CCK8 solution without drug solution.

### 2.5. Apoptosis Assay

Cells in culture were harvested, washed with cold PBS, and resuspended in 400 µL Annexin binding buffer. Thereafter, 5 µL of annexin V-FITC and 5 µL of PI (BD Pharmingen; BD Biosciences) were added to each sample, and after mixing, the sample was incubated for 15 min in the dark. Stained samples were measured using flow cytometry (FCM) within 1 h. CytExpert software2.0 (Beckman CytoFLEX) was used to analyze the FCM data.

### 2.6. 5-Ethynyl-2-Deoxyuridine (EdU) Assay

EdU staining was performed using the BeyoClick™ EdU-488 kit (Beyotime) according to the manufacturer’s protocol. The MM cells were inoculated into 96-well plates at a density of 5 × 10^3^ cells/well and treated with 10 μM EdU at 37 °C for 2 h. Cells were then subjected to a fixation step in PBS with 3% paraformaldehyde and a permeabilization step with 0.5% Triton X-100 at room temperature. The fixative was removed, and the cells were washed with PBS containing 3% BSA. Subsequently, the MM cells were incubated in a click additive solution and protected from light. The stained samples were analyzed using flow cytometry. CytExpert software (Beckman CytoFLEX) was used to analyze the FCM data.

### 2.7. ATP Assay

The ATP assay was performed using an ATP Assay Kit (Beyotime) according to the manufacturer’s protocol. Cells were collected and washed three times with PBS, followed by lysis with lysis solution. At the same time, gradient concentrations of ATP standards were prepared with the fresh lysate, and 100 μL of the samples to be tested and the diluted gradient ATP standards were added to the assay plate, followed by the addition of 100 μL of the ATP assay working solution, and the absorbance values of each group of cells were measured at 28 °C for 5 min. Finally, the ATP assay was calibrated using the protein quantification results in each group of cells.

### 2.8. Reactive Oxygen Species (ROS) Assay

The ROS assay was performed using an ROS Assay Kit (Beyotime) according to the manufacturer’s protocol. Cells were inoculated in 12-well plates at 2 × 10^6^ cells per well and pre-incubated in an incubator at 37 °C with a 5% CO_2_ atmosphere for 24 h. The cells were grouped for intervention after plastering. After incubating with the fluorescent probe 2,7-dichlorofluorescein diacetate (DCFH-DA), the cells were collected and detected using flow cytometry, according to the reactive oxygen species detection kit.

### 2.9. Mitochondrial Mass Assay

We used a flow cytometry-based method to assess mitochondrial mass using Mito Tracker Red CMXRos (Beyotime). Cells were washed twice with PBS and stained with 50 nM Mito Tracker Red CMXRos working solution for 30 min at 37 °C. At the end of the incubation period, cells were centrifuged at 1000× *g* for 5 min, the supernatant was discarded, and fresh cell culture solution was preincubated at 37 °C to resuspend the cells. Fluorescence was analyzed using the CytExpert software (Beckman CytoFLEX).

### 2.10. Measurement of Mitochondrial Oxygen Consumption Rate (OCR)

Cells were plated on 96-well cell culture plates and incubated overnight in a CO_2_ incubator at 37 °C, after which 150 μL of fresh medium was added to each well and incubated in a CO_2_ incubator at 37 °C for 30 min. Extracellular O_2_ depletion reagent (10 μL) (Ab197243, Abcam, Cambridge, UK) was added along with two drops of pre-warmed, high-sensitivity mineral oil. Fluorescence (EX 380 ± 40 nm, EM 615 ± 10 nm) was measured every 6 min for 90 min at 37 °C using a multi-detection microplate reader (Spectramax iD5, Molecular Devices, SAN Jose, CA, USA).

### 2.11. Glycolysis Assay

Glycolysis assays were performed according to the instructions provided in the Glycolysis Assay Kit (Ab197244; Abcam, Cambridge, UK). This assay measures extracellular acidification due to lactate production during glycolysis. Cells were incubated overnight in 96-well plates, as instructed, and then cleared from CO_2_ for 3 h at 37 °C in a CO_2_ free incubator. Next, 10 μL of glycolysis assay reagent containing a cell-impermeable pH-sensitive fluorophore and 150 μL of respiration buffer were added. Cells were then exposed to hypoxia, and the fluorescence (Ex 380 ± 40 nm Em 615 ± 10 nm) was measured every 6 min for 90 min at 37 °C using a multi-detector enzyme marker (SpectraMax iD5, Molecular Devices, USA). 

### 2.12. SiRNA Transfection to Knock down Pim-2

The MM cells were inoculated into 6-well plates, and approximately 2 mL of antibiotic-free medium was added to each well so that the cell density at the time of transfection was between 40% and 60%. Next, 4 μL/well of the Lipo 3000 transfection reagent was diluted with 200 μL of serum-free medium, mixed, and left for 5 min at room temperature. Eight microliters of the designed siRNA primer PIM2-Homo-525 (GenePharma), as well as siRNA GAPDH (positive control) and siRNA NC (negative control), was added to 200 μL of serum-free medium. The mixture was incubated for 20 min at room temperature before mixing with transfection reagents to form transfection complexes. The siRNA transfection reagent mixture was added to the cell culture plate, gently mixed, and incubated in a CO_2_ incubator. The proteins were extracted after 72 h of transfection for subsequent experiments.

### 2.13. Western Blotting Analysis

Cells were lysed with RIPA (Sigma-Aldrich, St. Louis, MO, USA) lysis buffer, and the supernatant was collected by centrifugation, followed by the addition of SDS loading buffer and boiling at 95 °C for 15 min. The cooled protein samples were added to polyacrylamide gels for electrophoretic separation. The separated protein samples in the gel were subsequently transferred to a PVDF membrane, and the antibodies were added after blocking in 5% nonfat milk for 1 h. Next, the membrane was washed thrice with TBST (Solarbio, Beijing, China) and incubated for 1 h with a secondary antibody at room temperature, followed by three TBST washes. The final results were observed by adding a luminescence solution and using an ECL chemiluminescence system.

The primary antibodies used were Pim-2 (#4730), AMPK-α (#5831S), and *p*-AMPK-α (#2535), which were purchased from Cell Signaling Technology (Danvers, MA, USA). LKB1 (ab199970) was purchased from Abcam (Cambridge, UK). The secondary antibody (#7074) was purchased from Cell Signaling Technology.

### 2.14. Statistical Analysis

We used the R package maxstat (maximally selected rank statistics with several *p*-value approximations, version: 0.7-25) to calculate the optimal cut-off value of Pim-2, setting the minimum grouping sample size to be greater than 25% and the maximum sample size grouping. The patients were divided into two groups with high and low expression of Pim-2, and the prognostic differences between the two groups were further analyzed using the Survfit function of the R package survival, and the significance of the prognostic differences between the samples of different groups was evaluated using the log-rank test method. We evaluated the prognostic significance of each gene using lasso Cox regression analysis by integrating survival time, survival status, and gene expression data using the R package survival. Three biological replicates were used for the metabolomic analysis. A Student’s *t*-test was performed using GraphPad Prism 7.0 software (GraphPad Software Inc., La Jolla, CA, USA). Statistical significance was set at *p* < 0.05. 

## 3. Results

### 3.1. High Expression of Pim-2 Kinase Correlates with Poor Prognosis and Genes Involved in Energy Metabolism in MM Patients

**Figure 1:**
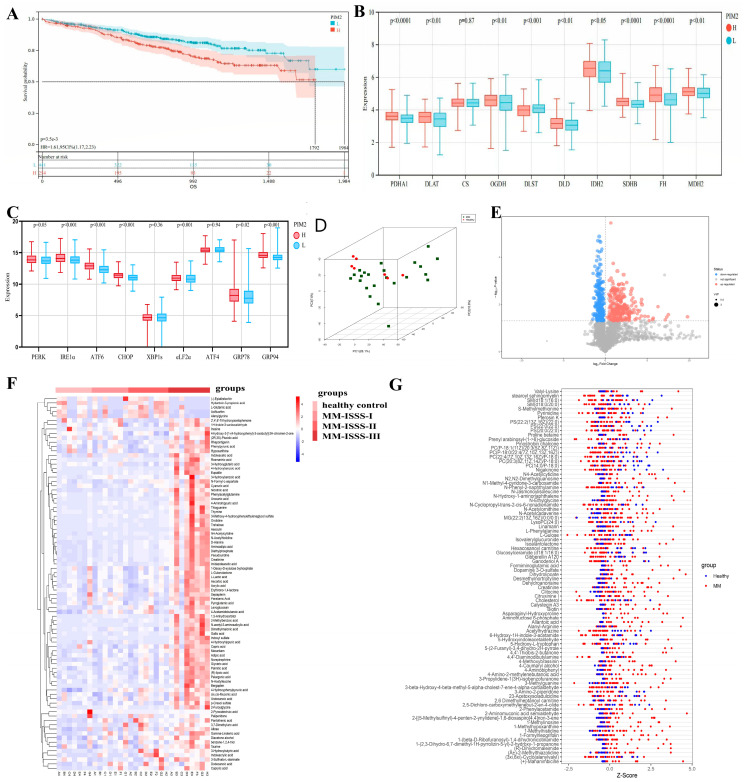
High expression of Pim-2 kinase correlates with poor prognosis and genes involved in energy metabolism and endoplasmic reticulum stress in MM patients. (**A**). Survival curves of MM patients by subgrouping according to Pim-2 expression. L means MM patients with low expression of Pim-2. (**B**). Association of pim2 expression levels with gluconeogenesis-related genes. L means MM patients with low expression of Pim-2. H means MM patients with high expression of Pim-2. (**C**). Association of pim2 expression levels with endoplasmic reticulum stress-related genes. (**D**). A 3D score scatter plot of a PCA model for group MM vs. HC. Samples are all at 95% confidence intervals. (**E**). Most metabolites of MM patients were increased. Significantly upregulated metabolites are depicted in red, significantly downregulated metabolites in blue, and non-significantly differential metabolites in gray. (**F**). The concentration of most metabolites in high-risk MM patients is high. The abscissa of the plot represents different experimental groupings, the ordinate represents the differential metabolites contrasted by the group, the color block at different positions represents the relative expression of the metabolite at the corresponding position, red indicates that the substance is highly expressed in the group where it is located, and blue indicates that the substance is lowly expressed in the group where it is located. (**G**). Metabolite content in MM patients was significantly increased. The abscissa represents the Z value, the ordinate represents the differential metabolites, and the dots in different colors represent samples in different groups.

We downloaded the available data from the Multiple Myeloma Research Foundation (MMRF, CoMpass) database, which includes RNA gene expression data from 731 patients with NDMM. We divided the patients into two groups with high and low Pim-2 expression and further analyzed the prognostic differences between the two groups. We observed a significant prognostic difference (*p* < 0.001), which suggested that high expression of Pim-2 kinase in MM patients was associated with poor prognosis (Figure 1A). Pim-2 is known to play an important role in aerobic glycolysis and tumor development. Therefore, we analyzed the cumulative expression levels of specific genes related to the gluconeogenic and endoplasmic reticulum stress pathway by grouping them according to Pim-2 expression levels. We found that gluconeogenesis and endoplasmic reticulum stress-related gene expression levels were elevated in patients with high Pim-2 expression levels (Figure 1B,C, Table 2). This implies that Pim-2 may be involved in the glyccometabolic process and endoplasmic reticulum stress in patients with MM.

Next, we performed a non-targeted metabolomic study of bone marrow supernatants from 30 MM patients and 20 healthy controls by LC-MS assay. Figure 1C,D show that there were significant differences among the HC, MM-ISSS-I, MM-ISSS-II, and MM-ISSS-III groups. Using the results from HC as a reference, the differences in metabolite changes in patients with MM were plotted in a volcano plot (Figure 1E). The results showed that most metabolite levels were upregulated in MM patients, indicating the presence of metabolic abnormalities in patients with MM. Hierarchical clustering analysis identified a high concentration of most metabolites in high-risk MM patients, which could distinguish these patients from HC and lower-risk MM patients (Figure 1F), suggesting that hypermetabolism may be associated with a poor prognosis of MM. This feature is also illustrated in Figure 1G, which shows that the metabolites were significantly upregulated in MM patients. These data indicate metabolic abnormalities in patients with MM, which may be related to the poor prognosis of MM.

### 3.2. Pim-2 Kinase Inhibitors Inhibit Glycometabolism in MM

**Figure 2:**
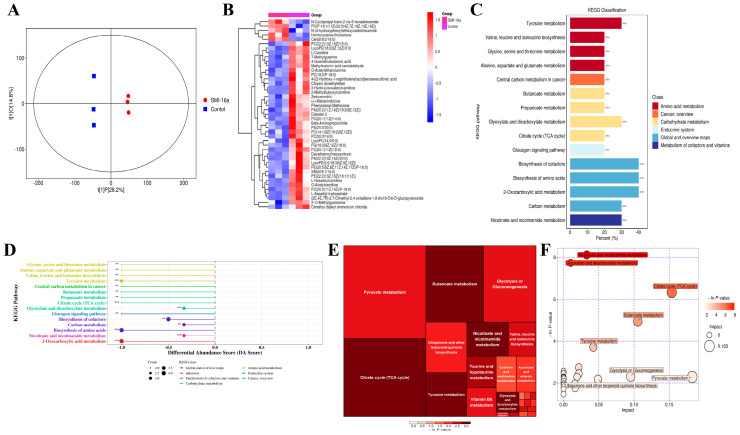
Pim-2 kinase inhibitors inhibit glycometabolism in MM. (**A**). Score scatter plot of OPLS-DA model for group SMI-16a vs. Control. Each scatter represents a sample, and scatter shape and color indicate different experimental groupings, with a greater lateral distance between samples illustrating greater group differences and a closer longitudinal distance illustrating better within group reproducibility. (**B**). Heatmap of hierarchical clustering analysis for SMI-16a vs. Control groups. (**C**). KEGG classification for group SMI-16a vs. Control. The abscissa represents the number of differential metabolites annotated under a certain pathway as a percentage of all the number of differential metabolites annotated, and the ordinate represents the enriched KEGG metabolic pathway names. (**D**). In the SMI-16a treatment group, glucose metabolism, lipid metabolism, and amino acid metabolism were all downregulated. The abscissa in the figure represents the differential abundance score (DA score) and the ordinate represents the KEGG metabolic pathway name. Dots distributed to the right of the middle axis and longer line segments indicate a greater tendency for the overall expression profile of the pathway to be upregulated; dots distributed to the left of the middle axis and longer line segments indicate a greater tendency for the overall expression profile of the pathway to be downregulated. (**E**). Glycometabolism plays an important role in MM cells. Pathway analysis for group SMI-16a vs. Control. Each square in the rectangular tree diagram represents a metabolic pathway, and the square size indicates the impact factor size of that pathway in the topological analysis. The square color indicates the *p*-value of the enrichment analysis, and the darker the color, the smaller the *p*-value and the more significant the enrichment. (**F**). Different metabolites were enriched in glycolysis or gluconeogenesis. Each bubble in the bubble plot represents a metabolic pathway, and the abscissa on which the bubble is located and the bubble size represent the effect factor size of that pathway in topology analysis, and the larger the effect factor, the larger the size. The ordinate where the bubble is located and the bubble color indicate the *p*-value of the enrichment analysis, and a darker color of the *p*-value indicates significant enrichment. ** *p* < 0.001; *** *p* < 0.0001.

Based on these findings, we aimed to determine the specific role of Pim-2 in the regulation of energy metabolism in MM patients. Therefore, we used LC-MS/MS to detect MM cell metabolites. The heatmap shows the main differential metabolites between the control and Pim-2 kinase inhibitor-treated MM cells (Figure 2A,B), indicating that the inhibition of Pim-2 leads to a significant reduction in metabolites. Enrichment analysis of metabolites revealed that several important metabolic pathways were perturbed in the SMI-16a-treated group. Metabolites, such as glucose, lipids, and amino acids, were all downregulated, resulting in the reduction of related metabolites and ATP. Further metabolic pathway analysis revealed that the differential metabolites were enriched in the “glycolytic or glycoisomeric” pathway in both positive and negative ion modes. Combined with the above findings, we focused on exploring the effects of Pim-2 on MM glycometabolism.

### 3.3. Pim-2 Kinase Inhibitor Participates in the Regulation of MM Cell Proliferation and Apoptosis

**Figure 3:**
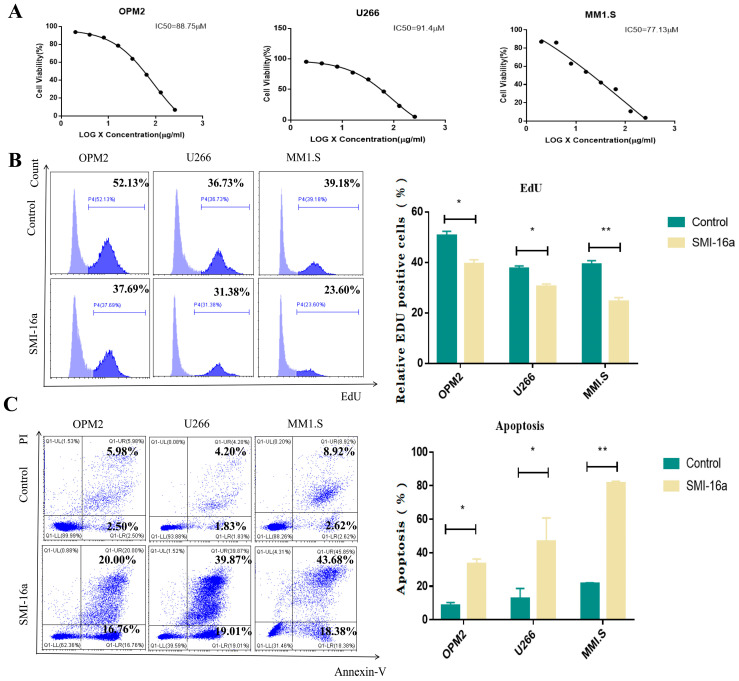
SMI-16a inhibited cell proliferation and induced cell apoptosis in MM cells. (**A**). Cell viability of MM cell lines treated with increasing concentrations of SMI-16a for 48 h. (**B**). EdU assay for viability of MM cells after SMI-16a treatment (*n* = 3 for each group). (**C**). The apoptosis assessment using propidium iodine (PI) and Annexin V detection by flow cytometry at 48 h of treatment (*n* = 3 for each group). * Indicates a significant difference compared with the HC group, *p* < 0.05. ** Indicates a significant difference compared with the HC group, *p* < 0.001.

To elucidate how Pim-2 participates in the development of MM, we verified whether inhibition of Pim-2 in MM cells could affect MM cell proliferation. First, we examined the dose-dependent effects of Pim-2 kinase inhibitors on MM cell proliferation using the CCK-8 assay. SMI-16a, a potent inhibitor of Pim-2 kinase, effectively attenuated the tumorigenic capacity of many human malignancies. In this study, we used SMI-16a to inhibit Pim-2 kinase in MM cells. The IC50 values of OPM2 cells, U266 cells, and MM1.S cells were 88.75, 91.4, and 77.13 μM, respectively (Figure 3A). Exposure to SMI-16a at concentrations of 100 µM or higher inhibited the growth of almost all tumor cells, indicating its significant anti-proliferative activity. Next, we conducted an EdU admixture test to assess the effect of Pim-2 kinase inhibitors on cell viability. Each cell line was treated at different concentrations according to the IC50 of the drug. The percentage of EdU-positive cells was lower in the SMI-16a-treated group than in the control group (Figure 3B). These data indicate that the Pim-2 kinase inhibitor had a significant inhibitory effect on MM cell proliferation.

We next assessed the ability of Pim-2 kinase inhibitors to induce apoptosis in MM cells. We used PI/Annexin V to double-stain MM cells and analyzed the apoptosis rate using flow cytometry. The percentage of apoptotic cells increased in a dose-dependent manner 48 h after SMI-16a treatment (Figure 3C). These results suggested that Pim-2 kinase inhibitors induce apoptosis in MM cells.

### 3.4. Inhibition of Pim-2 Reduces Cellular Glycometabolism and Energy Production

**Figure 4:**
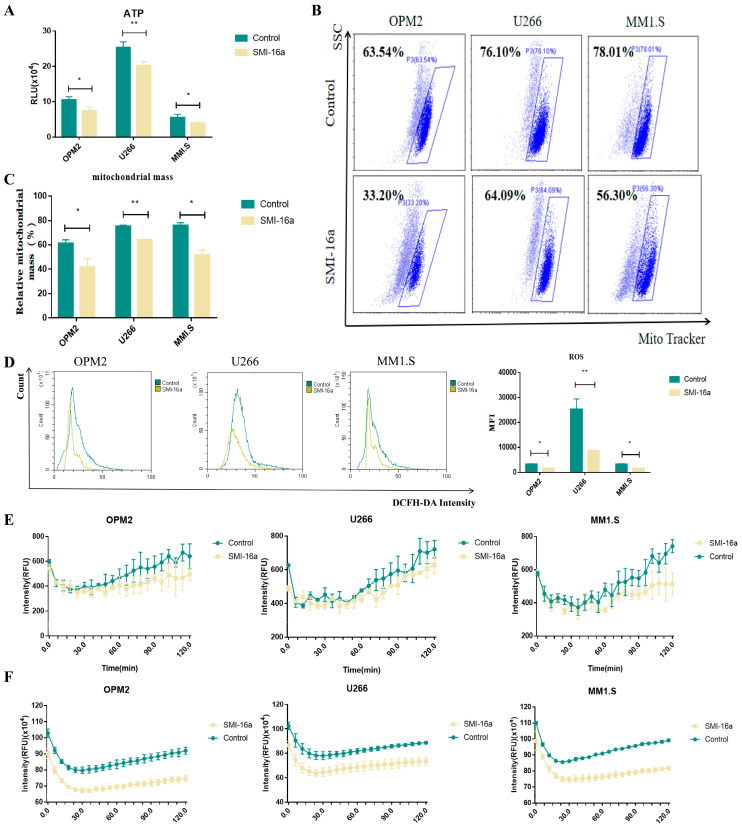
Pim-2 is involved in energy metabolism through participation in aerobic oxidation and glycolysis. (**A**). Detection of ATP production levels in different treatment groups. In different MM cells, ATP levels were lower in the SMI-16a-treated group than in the control group. (**B**). The detection of cellular mitochondrial mass after MM cell lines were treated with SMI-16a for 48 h. (**C**). The total percentage of mitochondrial mass in different cells. (**D**). Total percentage of ROS in different cells. (**E**). Oxygen consumption analysis. Control and SMI-16a groups were measured by fluorescence spectroscopy over 90 min and compared for fluorescence emission. (**F**). Glycolysis assay measured as cytoplasmic acidification. Glycolysis was measured in Control and SMI-16a groups by fluorescence emission over 90 min. Each group of experiments was repeated 3 times. * Indicates a significant difference compared with the HC group, *p* < 0.05. ** Indicates a significant difference compared with the HC group, *p* < 0.001.

Next, we evaluated the state of cells in terms of oxygen consumption and glycolysis. The results showed that Pim-2 inhibition led to a decrease in ATP production (Figure 4A), indicating that energy production was indeed induced by Pim-2. MitoTracker is a fluorescent dye that specifically marks the mitochondria and can be used to visualize and quantify these organelles in the cell. We found that mitochondrial mass was decreased in the SMI-16a-treated groups compared with that in the control group in different MM cell lines (Figure 4B,C). Next, we treated MM cells with SMI-16a for 48 h and measured the ROS levels. As shown in Figure 4D, the ROS level in the treated group decreased. This suggests that the Pim-2 kinase inhibitor may inhibit ROS production from mitochondria. We thus assessed the ability of the cells to consume O_2_. The results of the oxygen consumption study showed that the mitochondrial function of MM cells treated with SMI-16a also tended to be reduced as compared to that of the control cells (Figure 4E). These data illustrate that the inhibition of Pim-2 blocks MM cells from obtaining energy via mitochondrial aerobic oxidation. To detect aerobic glycolysis, which is the main mechanism by which MM cells produce energy, we measured the extracellular acidification rate (ECAR). The results showed that ECAR levels also decreased after Pim-2 inhibition compared to levels in the control (Figure 4F). This suggests that Pim-2 may promote aerobic glycolysis to generate more ATP to meet the energy demands of MM cell development.

### 3.5. Pim-2 Negatively Regulates PKM2

**Figure 5:**
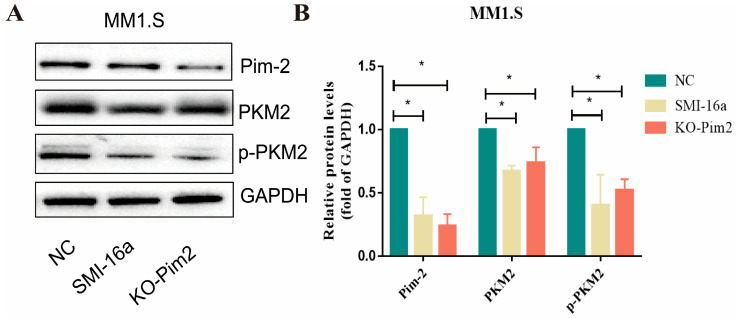
Pim-2 kinase inhibitors decrease PKM2 expression and phosphorylation. (**A**). Western blot analysis showed that the expression of p-PKM2 and PKM2 was downregulated. (**B**). The histogram of quantitative analysis of PKM2, p-PKM2, and Pim-2 (based on fold change of glyceraldehyde-3-phosphate dehydrogenase [GAPDH]). * Indicates a significant difference compared with the HC group, *p* < 0.05.

Numerous studies in recent years have shown that the pyruvate kinase 2 (PKM2) isoform of pyruvate kinase is a key regulator of the Warburg effect [17]. PKM2 promotes transcription of target genes such as GLUTs, LDH-A, and HIF-1α-targeted expression of VEGF-A, thereby promoting cancer cell growth and positive feedback regulation of glycolysis [18]. We verified the regulatory role of PKM2 in Pim-2 inhibition of glucose metabolism by knocking down Pim-2 in MM1.S cells with si-RNA and a Pim-2 kinase inhibitor. Western blotting results showed a significant reduction in PKM2 and PKM2 phosphorylation levels in Pim-2-silenced cells (Figure 5). Combined with previous data analysis, we speculate that Pim-2 silencing may reduce the glycolysis of MM cells by inhibiting PKM2 phosphorylation.

## 4. Discussion

Pim-2 kinase exerts its oncogenic effects by phosphorylating target proteins involved in cell cycle and proliferation [19,20,21]. Pim-2 kinase phosphorylates BAD at serine 112 and reverses BAD-induced cell death [22]. Pim-2 kinase is involved in the transduction of a variety of cellular signaling pathways, thereby promoting tumorigenesis, particularly in blood tissues. Newly developed Pim-2 kinase inhibitors are currently being tested in clinical trials [11,23,24], and these compounds, especially in combination with other drugs, have shown some success in MM treatment [23,25,26,27,28]. However, the mechanism of action of these treatments has not yet been fully elucidated.

In this study, we found that the level of Pim-2 increased in MM patients and was related to energy metabolism, which participated in the cell-cycle progression of myeloma cells, as indicated by mass spectrometry-based untargeted metabolomics analysis. Furthermore, inhibitors of Pim-2 kinase disrupted glycolytic flux and oxidative phosphorylation, thus inhibiting cell growth and ATP production in MM cells. We also demonstrated that Pim-2 kinase inhibitors regulated PKM2 involved in energy metabolism. Previous studies have demonstrated that PKM2 is the key rate-limiting enzyme in the Warburg effect as well as the rate-limiting enzyme in the final step of glycolysis in tumor cells and plays a key role in glycolysis [29]. Since PIM2 is a protein serine/threonine kinase and is a known phosphorylated protein, we speculate that PKM2 may be a substrate for PIM2. Furthermore, a previous study showed that Pim kinase directly increases the levels of PKM2, a key enzyme that regulates cellular metabolism and produces ATP [30]. In this study, we also confirmed that Pim-2 kinase inhibitors inhibit PKM2 phosphorylation and decrease PKM2 levels, which leads to a decrease in ATP. In conclusion, these results suggest that the metabolic regulation of Pim-2 kinase is one of the pathways involved in the proliferation of myeloma cells. In fact, we found many possible mechanisms for Pim-2, but we have only studied PKM2 so far, and other genes need to be studied in depth.

For other cancers, Pim-2 can promote glycolysis in some tumor cells by directly binding or changing the phosphorylation of PFKFB3 in breast cancer [31], PKM2 in HEK293T cells [32], and AMPKα in endometrial cancer [33]. Pim-2 activates glucose utilization and aerobic glycolysis in colorectal cancer cells and increases energy production. Knockdown of Pim-2 resulted in reduced cellular energy production and increased susceptibility to apoptosis [34].

Altered cellular metabolism in tumor cells is common and can manifest as abnormalities in mitochondrial function, thus allowing them to escape apoptosis [35]. Polarized M2 macrophages have been found to regulate tumor cell glycolysis through the secretion of interleukin-6 [36]. Altered adaptive energy metabolism in myeloma cells has also been correlated with resistance to proteasome inhibitors [37,38]. PRL-3, a cytokine-inducible oncogenic phosphatase, promotes glucose uptake and lactate excretion and increases glycine decarboxylase levels. The mRNAs of related proteins are associated with the expression of PRL-3 in primary myeloma cells [39]. FOXM1 is a positive regulator of myeloma cell metabolism and strongly influences the bioenergetics pathways of glycolysis and oxidative phosphorylation. The FOXM1 inhibitor NB73 slowed myeloma progression both in vitro and in vivo [40]. Knockdown of HSP60 or use of HSP90 inhibitors in myeloma cells results in the inhibition of proliferation, reduced mitochondrial mass, and weaker metabolic activity [40,41].

## 5. Conclusions

In summary, Pim-2 kinase regulated MM cell cycle, proliferation, and metabolism; acted on osteoblasts to regulate the development of bone disease; and modulated the immune microenvironment, highlighting that it is a potential therapeutic target in MM. Our study establishes that Pim-2 kinase can be involved in the development of MM by regulating MM energy metabolism.

## Figures and Tables

**Table 1:** Characteristics of the patients.

Characteristics	MM (*n* = 23)	Control (*n* = 30)	*p* Value
Age	64.69 ± 8.63	47.57 ± 13.62	0.1079
Female gender (%)	13 (56.52)	4 (57.14)	0.9778
Hemoglobin	68 ± 17.22	89.26 ± 30.99	0.7342
Serum calcium	2.26 ± 0.16	2.38 ± 0.36	0.3823
Creatinine	59.43 ± 24.90	214.78 ± 230.08	<0.0001
Albumin	37.17 ± 2.40	32.91 ± 5.80	0.0934
Lactate dehydrogenase	187.33 ± 54.42	223.11 ± 142.89	0.5566

**Table 2:** Relative expression levels of genes related to glycometabolism.

Label	High Pim-2 Expression Group (Mean ± Std)	Low Pim-2 Expression Group (Mean ± Std)	*p* Value
PDHA1	1 (3.61 ± 0.39)	0 (3.45 ± 0.39)	<0.001
DLAT	1 (3.49 ± 0.55)	0 (3.33 ± 0.66)	<0.001
CS	1 (4.42 ± 0.36)	0 (4.44 ± 0.37)	0.87
OGDH	1 (4.55 ± 0.61)	0 (4.38 ± 0.73)	<0.001
DLST	1 (3.95 ± 0.46)	0 (4.08 ± 0.48)	<0.001
DLD	1 (3.17 ± 0.45)	0 (3.06 ± 0.48)	<0.001
IDH2	1 (6.49 ± 0.71)	0 (6.32 ± 0.84)	<0.05
SDHB	1 (4.51 ± 0.35)	0 (4.35 ± 0.34)	<0.001
FH	1 (4.94 ± 0.67)	0 (4.59 ± 0.68)	<0.001
MDH2	1 (5.12 ± 0.40)	0 (5.02 ± 0.45)	<0.001

## Data Availability

All datasets that the conclusions of the paper rely on are available to readers and deposited in publicly available repositories.

## References

[1] Keane N.A., Reidy M., Natoni A., Raab M.S., O’Dwyer M. (2015). Targeting the Pim kinases in multiple myeloma. Blood Cancer J..

[2] Liu Z., Han M., Ding K., Fu R. (2020). The role of Pim kinase in immunomodulation. Am. J. Cancer Res..

[3] Szydlowski M., Garbicz F., Jablonska E., Gorniak P., Komar D., Pyrzynska B., Bojarczuk K., Prochorec-Sobieszek M., Szumera-Cieckiewicz A., Rymkiewicz G. (2021). Inhibition of PIM Kinases in DLBCL Targets MYC Transcriptional Program and Augments the Efficacy of Anti-CD20 Antibodies. Cancer Res..

[4] Zhang X., Song M., Kundu J.K., Lee M.H., Liu Z.Z. (2018). PIM Kinase as an Executional Target in Cancer. J. Cancer Prev..

[5] Wang Y., Xiu J., Ren C., Yu Z. (2021). Protein kinase PIM2: A simple PIM family kinase with complex functions in cancer metabolism and therapeutics. J. Cancer.

[6] Cowan A.J., Green D.J., Kwok M., Lee S., Coffey D.G., Holmberg L.A., Tuazon S., Gopal A.K., Libby E.N. (2022). Diagnosis and Management of Multiple Myeloma: A Review. JAMA.

[7] Kim S.D., Kim H.N., Lee J.H., Jin W.J., Hwang S.J., Kim H.H., Ha H., Lee Z.H. (2013). Trapidil, a platelet-derived growth factor antagonist, inhibits osteoclastogenesis by down-regulating NFATc1 and suppresses bone loss in mice. Biochem. Pharmacol..

[8] Paíno T., Garcia-Gomez A., González-Méndez L., San-Segundo L., Hernández-García S., López-Iglesias A.-A., Algarín E.M., Martín-Sánchez M., Corbacho D., Ortiz-de-Solorzano C. (2017). The Novel Pan-PIM Kinase Inhibitor, PIM447, Displays Dual Antimyeloma and Bone-Protective Effects, and Potently Synergizes with Current Standards of Care. Clin. Cancer Res..

[9] Johrer K., Obkircher M., Neureiter D., Parteli J., Zelle-Rieser C., Maizner E., Kern J., Hermann M., Hamacher F., Merkel O. (2012). Antimyeloma activity of the sesquiterpene lactone cnicin: Impact on Pim-2 kinase as a novel therapeutic target. J. Mol. Med..

[10] Claudio J.O., Masih-Khan E., Tang H., Goncalves J., Voralia M., Li Z.H., Nadeem V., Cukerman E., Francisco-Pabalan O., Liew C.C. (2002). A molecular compendium of genes expressed in multiple myeloma. Blood.

[11] Lu J., Zavorotinskaya T., Dai Y., Niu X.H., Castillo J., Sim J., Yu J., Wang Y., Langowski J.L., Holash J. (2013). Pim2 is required for maintaining multiple myeloma cell growth through modulating TSC2 phosphorylation. Blood.

[12] Liu Z., Liu H., Yuan X., Wang Y., Li L., Wang G., Song J., Shao Z., Fu R. (2018). Downregulation of Pim-2 induces cell cycle arrest in the G0/G1 phase via the p53-non-dependent p21 signaling pathway. Oncol. Lett..

[13] Morishita D., Katayama R., Sekimizu K., Tsuruo T., Fujita N. (2008). Pim kinases promote cell cycle progression by phosphorylating and down-regulating p27Kip1 at the transcriptional and posttranscriptional levels. Cancer Res..

[14] Yao R., Xie Y., Sun X., Zhang M., Zhou J., Liu L., Gao J., Xu K. (2020). Identification of a Novel c-Myc Inhibitor 7594-0037 by Structure-Based Virtual Screening and Investigation of Its Anti-Cancer Effect on Multiple Myeloma. Drug Des. Dev. Ther..

[15] Abe M. (2014). Myeloma bone disease. Clin. Calcium.

[16] Hiasa M., Teramachi J., Oda A., Amachi R., Harada T., Nakamura S., Miki H., Fujii S., Kagawa K., Watanabe K. (2015). Pim-2 kinase is an important target of treatment for tumor progression and bone loss in myeloma. Leukemia.

[17] Luo W., Semenza G.L. (2012). Emerging roles of PKM2 in cell metabolism and cancer progression. Trends Endocrinol. Metab..

[18] Luo W., Hu H., Chang R., Zhong J., Knabel M., O’Meally R., Cole R.N., Pandey A., Semenza G.L. (2011). Pyruvate kinase M2 is a PHD3-stimulated coactivator for hypoxia-inducible factor 1. Cell.

[19] Bachmann M., Hennemann H., Xing P.X., Hoffmann I., Moroy T. (2004). The oncogenic serine/threonine kinase Pim-1 phosphorylates and inhibits the activity of Cdc25C-associated kinase 1 (C-TAK1): A novel role for Pim-1 at the G2/M cell cycle checkpoint. J. Biol. Chem..

[20] Chen L.S., Balakrishnan K., Gandhi V. (2010). Inflammation and survival pathways: Chronic lymphocytic leukemia as a model system. Biochem. Pharmacol..

[21] Mondello P., Cuzzocrea S., Mian M. (2014). Pim kinases in hematological malignancies: Where are we now and where are we going?. J. Hematol. Oncol..

[22] Yan B., Zemskova M., Holder S., Chin V., Kraft A., Koskinen P.J., Lilly M. (2003). The PIM-2 kinase phosphorylates BAD on serine 112 and reverses BAD-induced cell death. J. Biol. Chem..

[23] Nair J.R., Caserta J., Belko K., Howell T., Fetterly G., Baldino C., Lee K.P. (2017). Novel inhibition of PIM2 kinase has significant anti-tumor efficacy in multiple myeloma. Leukemia.

[24] Ramakrishnan V.G., Kumar S.K. (2016). Inhibitors of the Cyclin-Dependent Kinase and PIM Kinase Pathways in the Treatment of Myeloma. Cancer J..

[25] Fujii S., Nakamura S., Oda A., Miki H., Tenshin H., Teramachi J., Hiasa M., Bat-Erdene A., Maeda Y., Oura M. (2018). Unique anti-myeloma activity by thiazolidine-2,4-dione compounds with Pim inhibiting activity. Br. J. Haematol..

[26] Koblish H., Li Y.L., Shin N., Hall L., Wang Q., Wang K., Covington M., Marando C., Bowman K., Boer J. (2018). Preclinical characterization of INCB053914, a novel pan-PIM kinase inhibitor, alone and in combination with anticancer agents, in models of hematologic malignancies. PLoS ONE.

[27] Adam K., Lambert M., Lestang E., Champenois G., Dusanter-Fourt I., Tamburini J., Bouscary D., Lacombe C., Zermati Y., Mayeux P. (2015). Control of Pim2 kinase stability and expression in transformed human haematopoietic cells. Biosci. Rep..

[28] Cortes J., Tamura K., DeAngelo D.J., de Bono J., Lorente D., Minden M., Uy G.L., Kantarjian H., Chen L.S., Gandhi V. (2018). Phase I studies of AZD1208, a proviral integration Moloney virus kinase inhibitor in solid and haematological cancers. Br. J. Cancer.

[29] He X., Du S., Lei T., Li X., Liu Y., Wang H., Tong R., Wang Y. (2017). PKM2 in carcinogenesis and oncotherapy. Oncotarget.

[30] Alquraishi M., Puckett D.L., Alani D.S., Humidat A.S., Frankel V.D., Donohoe D.R., Whelan J., Bettaieb A. (2019). Pyruvate kinase M2: A simple molecule with complex functions. Free. Radic. Biol. Med..

[31] Lu C., Qiao P., Sun Y., Ren C., Yu Z. (2021). Positive regulation of PFKFB3 by PIM2 promotes glycolysis and paclitaxel resistance in breast cancer. Clin. Transl. Med..

[32] Yu Z., Zhao X., Huang L., Zhang T., Yang F., Xie L., Song S., Miao P., Zhao L., Sun X. (2013). Proviral insertion in murine lymphomas 2 (PIM2) oncogene phosphorylates pyruvate kinase M2 (PKM2) and promotes glycolysis in cancer cells. J. Biol. Chem..

[33] Han X., Ren C., Yang T., Qiao P., Wang L., Jiang A., Meng Y., Liu Z., Du Y., Yu Z. (2019). Negative regulation of AMPKalpha1 by PIM2 promotes aerobic glycolysis and tumorigenesis in endometrial cancer. Oncogene.

[34] Zhang X.H., Yu H.L., Wang F.J., Han Y.L., Yang W.L. (2015). Pim-2 Modulates Aerobic Glycolysis and Energy Production during the Development of Colorectal Tumors. Int. J. Med. Sci..

[35] Zong W.X., Rabinowitz J.D., White E. (2016). Mitochondria and Cancer. Mol. Cell.

[36] Zhang Y., Yu G., Chu H., Wang X., Xiong L., Cai G., Liu R., Gao H., Tao B., Li W. (2018). Macrophage-Associated PGK1 Phosphorylation Promotes Aerobic Glycolysis and Tumorigenesis. Mol. Cell.

[37] Maiso P., Huynh D., Moschetta M., Sacco A., Aljawai Y., Mishima Y., Asara J.M., Roccaro A.M., Kimmelman A.C., Ghobrial I.M. (2015). Metabolic signature identifies novel targets for drug resistance in multiple myeloma. Cancer Res..

[38] Zaal E.A., Wu W., Jansen G., Zweegman S., Cloos J., Berkers C.R. (2017). Bortezomib resistance in multiple myeloma is associated with increased serine synthesis. Cancer Metab..

[39] Abdollahi P., Vandsemb E.N., Elsaadi S., Rost L.M., Yang R., Hjort M.A., Andreassen T., Misund K., Slordahl T.S., Ro T.B. (2021). Phosphatase of regenerating liver-3 regulates cancer cell metabolism in multiple myeloma. FASEB J..

[40] Cheng Y., Sun F., Thornton K., Jing X., Dong J., Yun G., Pisano M., Zhan F., Kim S.H., Katzenellenbogen J.A. (2022). FOXM1 regulates glycolysis and energy production in multiple myeloma. Oncogene.

[41] Wu X., Guo J., Chen Y., Liu X., Yang G., Wu Y., Tian Y., Liu N., Yang L., Wei S. (2020). The 60-kDa heat shock protein regulates energy rearrangement and protein synthesis to promote proliferation of multiple myeloma cells. Br. J. Haematol..

